# GNAS Mutations Identify a Set of Right-Sided, RAS Mutant, Villous Colon Cancers

**DOI:** 10.1371/journal.pone.0087966

**Published:** 2014-01-30

**Authors:** Ryan E. Fecteau, James Lutterbaugh, Sanford D. Markowitz, Joseph Willis, Kishore Guda

**Affiliations:** 1 Department of Pathology, Case Western Reserve University and Case Medical Center, Cleveland, Ohio, United States of America; 2 Case Comprehensive Cancer Center, Case Western Reserve University and Case Medical Center, Cleveland, Ohio, United States of America; 3 Department of Medicine, Case Western Reserve University and Case Medical Center, Cleveland, Ohio, United States of America; 4 Department of Genetics and Genome Sciences, Case Western Reserve University and Case Medical Center, Cleveland, Ohio, United States of America; 5 Division of General Medical Sciences-Oncology, Case Western Reserve University and Case Medical Center, Cleveland, Ohio, United States of America; Sanford Burnham Medical Research Institute, United States of America

## Abstract

The purpose of this study is to determine the genetic frequency of GNAS activating mutations in colorectal cancer and the corresponding pathology of GNAS mutant tumors. Oncogenic mutations in GNAS have been described in a number of neoplasms including those of the pituitary, kidney, pancreas, and, more recently, in colon cancer. To ascertain the frequency in colon cancer we employed a sensitive pyrosequencing platform for mutation detection of the R201C and R201H GNAS hotspots in tumor samples representing all clinical stages. We additionally assayed for KRAS and BRAF mutations as previous reports have shown that these often co-occur with activating GNAS mutations. Of the 428 colon tumors assayed, mutations in GNAS were present in 10 of the samples (2.3%), indicating this is a significant, albeit infrequent, mutation in colorectal tumors. Nine GNAS mutant tumors (90%) harbored concomitant activating mutations in either the KRAS or BRAF oncogene, which was significantly greater than the mutation frequency of these genes in the tumor population (56%, p<0.0305). All ten of the GNAS mutant tumors arose in the right (proximal) colon (p<0.007), and 7 of 8 reviewed cases exhibited a marked villous morphology. Taken together, these data indicate that GNAS mutant colon tumors commonly have synchronous mutations in KRAS or BRAF, are right-sided in location, and are associated with a villous morphology.

## Introduction

Colorectal tumorigenesis is characterized by a succession of genetic aberrations that transform the normal colonic epithelium into an invasive cancer [1]. We have previously defined a set of 140 genes that undergo recurrent somatic mutation in colorectal cancer (CRC), suggesting these are likely drivers of tumor progression [2], [3]. One of these genes was GNAS, in which we found recurrent mutations at codon 201, that altered the highly conserved Arg^201^ to either cysteine (R201C) or histidine (R201H) [2], [3]. GNAS is a known oncogene that was first described in growth-hormone secreting pituitary adenomas and has since been found to be mutated in a number of neoplasms, predominantly at the codon 201 hotspot [4]–[7]. GNAS codon 201 mutations are particularly frequent in intrapapillary mucinous neoplasias (IPMN) of the pancreas where 67% of cases are mutant [8]. The major product of the GNAS locus, the Gsα subunit of heterotrimeric G-proteins, acts to transduce signals from G-protein coupled recptors (GPCRs) to the effector enzyme adenylate cyclase in the G-stimulatory (Gs) pathway, leading to the production of cyclic AMP (cAMP). Both R201C and R201H mutations result in constitutive activation of Gsα and autonomous cAMP production [9], [10].

In IPMNs, GNAS mutations are frequently accompanied by mutations of KRAS, with 51% of GNAS mutant cases also bearing mutations in KRAS [8]. Activating mutations in KRAS, and to a lesser extent, its downstream effector BRAF, are frequent events in colon cancer. Moreover, full exome sequencing of colorectal cancer samples by our group and collaborators revealed coincident GNAS and KRAS mutations in a small cohort of colon tumors, indicating GNAS mutations in colon cancers might also often be accompanied by mutations in KRAS and/or BRAF [2].

Reported frequencies of GNAS activating mutations in CRC have differed among various groups, ranging from as little as 0.5% to 9% [2], [11]–[13]. In the present study, we employed a sensitive pyrosequencing platform to sequence the codon R201 mutational hotspot in a cohort of 428 sporadic colon tumors to ascertain a more precise frequency in CRC. We also assayed for mutations in KRAS and BRAF to determine the prevalence of coincident GNAS/KRAS or GNAS/BRAF mutations in our tumor cohort. Clinical and pathologic data was reviewed to determine if GNAS mutant tumors are associated with any unique clinical or morphologic phenotypes. Here we show that GNAS mutant colon tumors harbor recurrent KRAS or BRAF mutations, target the anatomic proximal “right-sided” colon, and exhibit a villous morphology.

## Materials and Methods

### Ethics Statement

The tumor sample accrual protocol entitled, “CWRU 7296: Colon Epithelial Tissue Bank”, was approved by the University Hospitals Case Medical Center Institutional Review Board for Human Investigation with the assigned UH IRB number 03-94-105. Under this protocol, discarded tissue was obtained through written informed consent from patients for research use.

### Tumor Specimens

Tumor specimens were obtained from a frozen archive that consisted of 428 unselected colorectal cancers without reported family history (hereafter referred to as sporadic colorectal adenocarcinomas) accrued under the above protocol. Clinical data was obtained and assembled through individual pathology case reports for each tumor. Microscopic review of tumor morphology for selected samples was performed by an anatomic pathologist (J.W). All samples were assayed for mutations in GNAS codon 201, KRAS codons 12, 13, 61, and 146, and BRAF codon 600 using both Sanger sequencing and pyrosequencing.

### DNA extraction and MSI testing

Genomic DNA extraction from tumor samples was performed using either a standard guanidine thiocyanate protocol [14] or the DNeasy Blood and Tissue Kit (QIAGEN). Testing for MSI status at genomic loci BAT26 and BAT40 was performed as previously described [15].

### Pyrosequencing

Pyrosequencing analysis of GNAS codon 201 was performed on all samples. Pyrosequencing assays were designed using the PSQ Assay Design software (QIAGEN, Chatsworth, CA) that included GNAS codon 201, KRAS codons 12 and 13, KRAS codon 61, KRAS codon 146, and BRAF codon 600. For each assay, one of the PCR primers was biotinylated at the 5′ end and purified using high performance liquid chromatography. Primer sequences are as follows. GNAS: For: 5′-biotin-TTGGCTTTGGTGAGATCCATTG-3′, Rev 5′- CACCTGGAACTTGGTCTCAAAGAT-3′, Seq 5′- TTCCAGAAGTCAGGACA-3′; KRAS codons 12 and 13: For 5′- TCGATGGAGGAGTTTGTAAATGA-3′, Rev 5′- biotin-TTCGTCCACAAAATGATTCTGA-3′, Seq 5′-CTTGTGGTAGTTGGAGC-3′; KRAS codon 61: For 5′- CAGACTGTGTTTCTCCCTTCTCA-3′, Rev 5′- biotin-TCCTCATGTACTGGTCCCTCATTG-3′, Seq 5′- ATATTCTCGACACAGCAG-3′; KRAS codon 146: For 5′-AGGCTCAGGACTTAGCAAGAAGTT-3′, Rev 5′-biotin-GCCCTCTCAAGAGACAAAAACAT-3′, Seq 5′-AATTCCTTTTATTGAAACAT-3′. BRAF codon 600: For 5′- TTCATGAAGACCTCACAGTAAAAA-3′, Rev 5′- biotin-CCACAAAATGGATCCAGACA-3′, Seq 5′- TGATTTTGGTCTAGCTACA-3′. All PCR reactions were performed using FastStart Taq (Roche) and primer concentrations of 0.2 uM. Cycling conditions included an initial denaturation step at 95 C for 4 min, and 49 cycles of 95 for 15 s, 54 C for 30 s, and 72 C for 20 s. Following PCR, amplification products were sequenced on a PyroMark MD pyrosequencing instrument (QIAGEN) and mutation analysis was conducted as previously described [16].

### Sanger Sequencing

Sanger sequencing was used to confirm all mutations detected by pyrosequencing analysis. Isolated genomic DNA from tumor samples was used for PCR amplification of regions encompassing codon 201 of GNAS, codons 12, 13, 61, and 146 of KRAS, and codon 600 of BRAF. Forward and reverse primers used for PCR amplification were tagged with a 5′ M13 forward (5′-GTAAAACGACGGCCAGT-3′) and 5′ M13 reverse (5′-CAGGAAACAGCTATGAC-3′) universal primer sequence, respectively. Primer sequences were as follows. GNAS: For 5′- GTTGGCAAATTGATGTGAGC-3′, Rev 5′- CCCTGATCCCTAACAACACAG-3′; KRAS codons 12 and 13: For 5′-TGGTGGAGTATTTGATAGTGTA-3′, Rev 5′- CATGAAAATGGTCAGAGAA-3′; KRAS codon 61: For 5′- TCCAGACTGTGTTTCTCCCT-3′, Rev 5′- AACCCACCTATAATGGTGAATATCT-3′; KRAS codon 146: For 5′-AGAAGCAATGCCCTCTCAAG-3′, Rev 5′-GGACTCTGAAGATGTACCTATGGTC-3′ BRAF codon 600: For 5′- TCATAATGCTTGCTCTGATAGGA-3′, Rev 5′-GGCCAAAAATTTAATCAGTGGA-3′. All reactions were carried out using 0.4 uM concentration of each primer and FastStart Taq polymerase (Roche, Indianapolis, IN). Cycling conditions for all primer pairs consisted of an initial denaturation at 95 C for 4 min followed by 39 cycles of 95 C for 30 s, 58 C for 30 s, 72 C for 30 s, and a final elongation at 72 C for 3 min.

### Statistical Analysis

Fisher's exact test was used to assess differences in the proportion of GNAS mutant tumors between classes of gender, ethnicity, KRAS/BRAF status, microsatellite stability status, clinical stage, and tumor location. A two-tailed P-value of less than 0.05 was considered significant.

## Results

### Frequency of GNAS Hotspot Mutations in Colorectal Cancer

**Figure 1:**
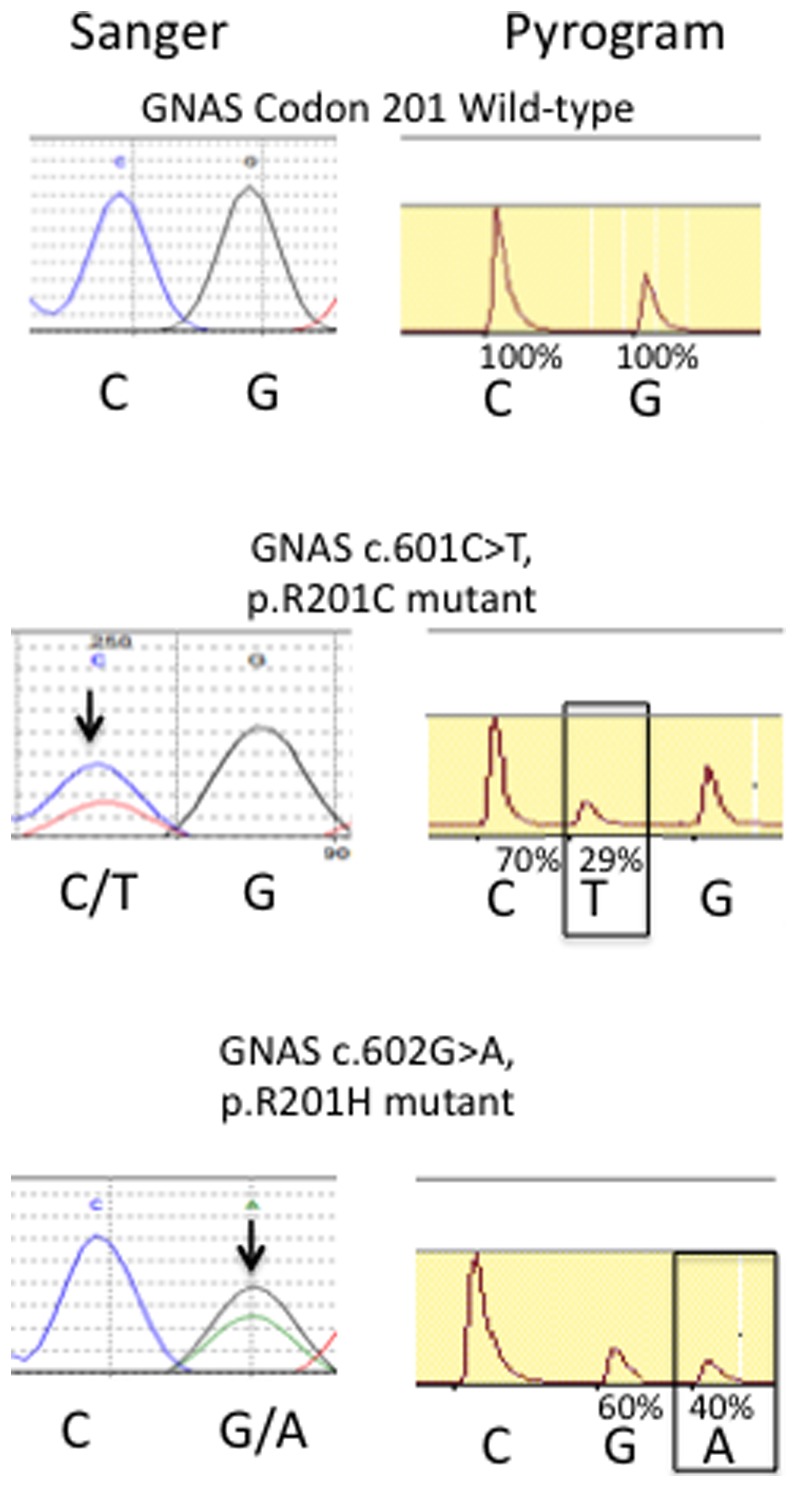
GNAS Codon 201 Mutations. Representative chromatograms (left) and pyrograms (right) of GNAS wild-type, GNAS R201C, and GNAS R201H mutations detected in colon cancer. Arrows in chromatograms show mutant, heterozygous peaks. Boxed peaks in pyrograms highlight mutant alleles. Percentages indicate allele frequencies calculated from pyrogram peak intensities.

Pyrosequencing detected activating mutations of GNAS codon 201 in ten of the 428 (2.3%) colorectal adenocarcinomas (Table 1). Each of these mutations was also validated using Sanger sequencing (Fig. 1). Of the ten GNAS codon 201 mutations detected, we identified seven p.R201H and three p.R201C amino acid substitutions, all of which were mutually exclusive (Table 2). Seven of these mutations detected arose among the 377 microsatellite stable tumors tested (1.9%), and three arose among the 41 tumors with microsatellite instability (7.7%). The increased frequency of GNAS mutations in microsatellite unstable tumors was of borderline statistical significance (P = 0.065). The mutation frequency at codon 201 of 0.0063 per diploid base pair is significantly higher than the background mutation rate of 1.2×10^−6^ mutations per base pair that typifies microsatellite stable colon cancers (P = 3E(−36)) [3].

**Table 1:** Tumor molecular and clinical characteristics according to GNAS mutation status.

GNAS Status	GNAS WT (%)	GNAS Mutant (%)	Total	P-Value
	418 (97.7%)	10 (2.3)	428	
**Tumor Location**				
Left	129 (30.9%)	0		
Right	195 (46.7%)	10 (100%)		
N/A	94 (22.5%)	0		**Left vs. Right**
**Total**	418	10	428	***(0.007*** *)*
**KRAS/BRAF Status**				
KRAS/BRAF WT	182 (43.5%)	1 (10%)		**KRAS/BRAF Mut vs. WT**
KRAS/BRAF Mut	236 (56.5%)	9 (90%)		
**Total**	418	10	428	***(0.0305)***
**Gender**				
Male	207 (49.5%)	5 (50%)		
Female	211 (50.5%)	5 (50%)		**Male vs. Female**
**Total**	418	10	428	(0.6128)
**Ethnicity**				
Caucasian	337 (80.4%)	8 (80%%)		**African American vs. Caucasian**
African Amer	71 (17.2%)	2 (20%)		
N/A	10 (2.4%)	0		
**Total**	418	10	428	(0.5452)
**Stage**				
Stage I&II	144 (34.4%)	3 (30%)		**Stage I&II vs. III&IV**
Stage 3&4	274 (65.6%)	7 (70%)		
**Total**	418	10	428	(0.5313)
**MMR status**				
MSS	369 (88.3%)	7 (70%)		
MSI	38 (9.1%)	3 (30%)		**MSS vs. MSI**
N/A	11 (2.6%)	0		(0.065)
**Total**	418	10	428	

Abbreviations: MMR, Mismatch Repair; MSS, Microsatellite Stable; MSI, Microsatellite instability.

**Table 2:** Clinical and molecular characteristics of GNAS mutant tumors.

Tumor #	Stage	GNAS Result	Kras Result	Braf Result	Location	MMR status	Gender	Ethnicity	Morphology
**1**	II	**R201H**	**G12D**	WT	**right**	MSS	Female	Caucasian	**Villous**
**2**	II	**R201H**	WT	**V600E**	**right**	MSS	Female	Caucasian	**Villous**
**3**	II	**R201C**	WT	**V600E**	**right**	MSI	Male	Caucasian	**Villous**
**4**	III	**R201C**	WT	**V600E**	**right**	MSI	Male	Caucasian	Signet
**5**	IV	**R201H**	**G12V**	WT	**right**	MSS	Female	Caucasian	**Villous**
**6**	IV	**R201H**	**G12V**	WT	**right**	MSS	Female	Afr Amer	**Villous**
**7**	IV	**R201H**	WT	WT	**right**	MSI	Male	Caucasian	N/A
**8**	IV	**R201H**	**G12V**	WT	**right**	MSS	Male	Caucasian	N/A
**9**	Met	**R201H**	**G13D**	WT	**right**	MSS	Female	Caucasian	**Villous**
**10**	Met	**R201C**	**A146T**	WT	**right**	MSS	Male	Afr Amer	**Villous**

Abbreviations: MMR, mismatch repair; Met, metastasis; Afr Amer, African American.

### GNAS mutations are associated with a villous morphology

**Figure 2:**
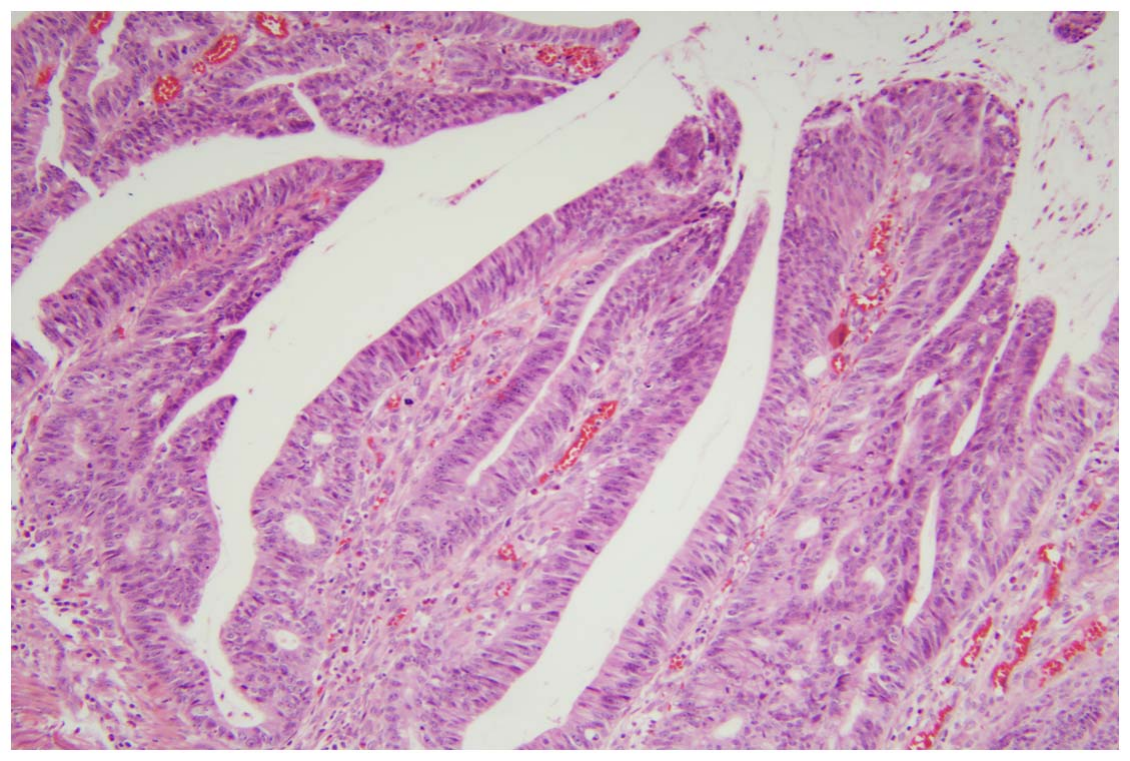
GNAS tumors are associated with a villous morphology. H&E Photomicrograph of a GNAS mutant, villous adenocarcinoma. The tumor has typical villous morphology with protruding papillae containing a fibrovascular core and lined with adenomatous epithelium.

Pathology review of GNAS mutant tumors revealed a prominent villous morphology in seven of eight (88%) cases available for review. In five of these cases, the GNAS mutant cancers arose in a contiguous villous adenoma. In two of these cases, the cancers themselves demonstrated a highly unusual villous architecture (Figure 2). Villous adenomas are a subtype of adenomatous polyp, accounting for approximately 5–15% of adenomas [17]. Villous cancers, however, are currently not recognized as a specific sub-classification of CRC, though studies suggest that villous adenocarcinomas may account for approximately 9% of CRC cases [18], [19]. Our results indicate that GNAS mutant tumors near exclusively arise in association with villous morphology present in either the cancer or the antecedent adenoma.

A recent study by Yamada and colleagues detected GNAS codon 201 hotspot mutations in 83% villous adenomas of the colon and rectum among Japanese patients [13]. We accordingly extended our mutation analysis to a small panel of villous, tubulovillous, and tubular adenomas to determine the frequency of GNAS mutations in a western adenoma cohort (Table 3). Overall, we found GNAS codon 201 mutations in 46% (6/13) of villous adenomas and in 15% (3/20) of tubulovillous adenomas, and in no tubular adenomas. Thus GNAS mutations in adenomas arise exclusively in the context of villous morphology. However, in the western population we characterized we find that villous adenomas divide molecularly into two approximately equal sized groups, one group comprised of villous adenomas that arise via the GNAS mutation pathway, and the other set of villous adenomas that arise through an alternative pathway.

**Table 3:** GNAS mutation frequency in adenomas.

Adenoma type	Count	GNAS Mutant (%)
Tubular	17	0
Tubulovillous	20	3 (15%)
Villous	13	6 (46%)

### GNAS mutant CRCs are predominantly KRAS/BRAF mutant and located in the proximal colon

Among IPMNs, GNAS mutations are frequently co-accompanied by mutations of KRAS. Among GNAS mutant colon cancers, we identified mutations in the KRAS and BRAF oncogenes in nine of the ten (90%) cases, a fraction that is significantly higher than the overall frequency of KRAS and BRAF mutations in the tumor cohort (P<0.0305, Fisher's exact test). The overall frequency of KRAS and BRAF mutations in our tumor population was approximately 56% (44% KRAS, 12% BRAF), which is in agreement with previous studies [20]. When examined for anatomical location within the colon, all ten GNAS mutant samples were found to be derived from primary cancers that originated in the proximal colon, between the cecum and splenic flexure, lesions commonly referred to as right-sided (P<0.007, Fisher's exact test). Included in our tumor cohort were both microsatellite stable (MSS) and microsatellite unstable (MSI) colon tumors, with MSI cases arising predominantly on the right side. Because three GNAS mutant tumors were also MSI tumors, it is possible that including MSI cancers skews the association of GNAS mutations with the proximal colon. However, when all MSI cancers are excluded from the analysis, the association of GNAS positive cancers with the proximal colon remains significant (P<0.044)

It is interesting to note that while all GNAS mutant cancers were right-sided, GNAS mutant adenomas were found throughout the colon (Table 4). No significant differences were observed in GNAS mutant tumors when analyzed for gender, ethnicity, clinical stage, or microsatellite status.

**Table 4:** Characteristics of GNAS mutant adenomas.

ID	GNAS status	Morphology	Location
Ad-1	R201H	TVA	right
Ad-2	R201H	TVA	right
Ad-3	R201H	TVA	right
Ad-4	R201C	VA	left
Ad-5	R201H	VA	transverse
Ad-6	R201H	VA	rectum
Ad-7	R201H	VA	rectum
Ad-8	R201C	VA	right
Ad-9	R201C	VA	left

Abbreviations: TVA, tubulovillous adenoma; VA, villous adenom.

## Discussion

In this study we find that GNAS mutations associate with a distinct subclass of colon cancers that is typified by location in the proximal colon, by having coincident KRAS or BRAF mutation, and by association with villous morphology. Although the frequency of GNAS mutant colon cancers is 2.3%, we find these tumors constitute a distinct molecular-pathologic subclass of colon cancer. To our knowledge, this analysis of 428 colon tuomrs is the most extensive analysis of these mutations that has been done. The 2.3% frequency of GNAS mutations in this cohort, is higher than that reported most recently by Idziaszczyk and colleagues in 2010, though less than initially observed in a previous smaller study by we and collaborators [2]. Intriguingly, GNAS mutant cancers are not found in the distal colon, whereas GNAS mutant villous adenomas arise throughout the colon, raising the possibility that GNAS mutant adenomas progress more rapidly to cancer in the proximal colon or are more likely to become symptomatic and detected when located in the distal colon. These questions will need to be further addressed experimentally to distinguish between these two possibilities.
